# A Review of Its Medical Aspects

**Published:** 1909-02-27

**Authors:** 


### THE POOR-LAW COMMISSION'S REPORT.

### A REVIEW OF ITS MEDICAL ASPECTS.

The Majority and Minority Reports of the Royal Commission on the Poor Laws and Relief of Distress cover 1,238 pages, about one-tenth of which deals with the question of medical relief, and only the parts referring to this are dealt with. The general recommendation of the Majority is that the present Boards of Guardians be abolished, and that "in future the members of the local authority should be largely nominated from amongst men and women of experience, wisdom, and unselfish devotion to the public good," and that "the new local authority should be known as the Public Assistance Authority, and the bodies which carry on its work in the smaller areas should be known as the Public Assistance Committees." The new authority is to be a Statutory Committee of the County or County Borough Councils. The Councils may appoint as to one-half from their own members, the other half to be appointed by the Councils from outside their number. For London certain modifications of this scheme are recommended.

### THE MAJORITY REPORT.

#### Development of the System of Medical Assistance to the Poor.

Poor-law medical relief, prior to 1834, was rather the outcome of practice than a creation of the law. The silence of early statutes is due in great measure to the reliance placed on voluntary association, in which hospitals and general dispensaries played a large part. The Act of 1834 did not specifically provide for such a system, and it was not till the Metropolitan Poor Act, 1867, established sick asylums and dispensaries in London and the Poor-law Amendment Act of 1868 empowered the Central Authority *inter alia* to order the provision of surgical and medical appliances in a workhouse, that the Legislature provided for a system of medical relief. There have been no enactments since then, and the system in existence is almost entirely based upon the orders of the Central Authority. This was the starting-point of modern development of Poor-law medical relief.

Such relief meant originally surgical and medical attendance and all medical and surgical appliances, but the Medical Relief Disqualification Removal Act, 1885, extended this into "all matters and things supplied by or on the recommendation of the medical officer." It was originally confined to cases of actual necessity or destitution, but destitution as applied to medical relief has come to mean inability to provide the treatment necessary, so the Poor Law has been obliged to provide for the sick on a scale continually advancing with the standard of care of the sick.

#### Indoor Medical Relief.

Since 1867 the principle of separate infirmary treatment for the indoor sick has been steadily pushed to the front in the Metropolis and the provinces, and an improvement made in the workhouse sick wards in many unions other than those which have provided infirmaries. In no direction is the improvement more marked than in nursing. Prior to 1865 the poor were chiefly nursed by paupers, and the only qualification prescribed for the paid nurse was ability "to read written directions upon medicines." By 1878-9, in all the sick asylums and separate infirmaries, the paid nurse had entirely superseded the pauper. In 1897 the pauper nurse was banished from the workhouse ward, and nurses and assistant nurses were to have practical experience in nursing. While in 1866 there were only 111 paid nurses in the metropolitan workhouses, in 1901 there were 1,246 trained nurses on the metropolitan nursing staff, 1,924 in the rest of England and Wales, and over 2,000 probationers.

The cost of treatment per head in metropolitan infirmaries has increased from £37 in 1878 to £50 in 1906. Capital expenditure per bed in the country is about £150, in London (owing to greater requirements) about £250. In staffing and equipment a great development has taken place, and in a number of the larger infirmaries the most delicate surgical operations are now performed by the medical staff.

The improvement has been such that the poor now gladly accept the benefits of treatment in the infirmaries, in spite of the investigation of their affairs by the Poor-law officials; district medical officers make more use of them than formerly, and relieving officers are always impressing on applicants for relief that the infirmary is not like the workhouse. All over the country the infirmaries attract persons of a higher station than formerly. The rise in the social condition of the patients can be seen by the payments for treatment made either by the patients themselves or by their relatives. At Mill Road, Liverpool, over £5,000 a year is collected from relatives. On the other hand, indoor medical relief, where still given in the workhouse, "is strongly deterrent to certain, and perhaps often the poorer and more self-respecting, members of the community, especially in country districts."

Apart from the increased attractiveness and efficiency, there are three forces which tend to the increased use of the infirmaries. Sanitary authorities have emphasised the institutional or segregative treatment of infectious diseases, but except in a few places they have not provided isolation hospitals for any but the highly infectious diseases. The infirmaries are the only institutions to which cases of erysipelas, puerperal fever, measles, whooping-cough, and chicken-pox can be sent for treatment. Secondly, increased pressure upon the accommodation of the general hospitals has led to a transference of cases from such hospitals to the Poor Law. Thirdly, in some parts of London and certain provincial districts general hospitals are either non-existent or too inaccessible.

#### Outdoor Medical Relief.

The system set up by the Poor-law Commissioners is, with a few exceptions, the system now in force. Unions are divided into districts, and a poor person must obtain an order from the Guardians, their Relieving Officer, or an Overseer. Permanently disabled, aged, and infirm paupers are placed on a "permanent list." Originally the remuneration of the medical officers was fixed by open competitive tender, a practice very soon abolished. Payment by fixed salary still largely prevails, with special fees for surgical operations and obstetrical work in terms of a scale originally fixed in 1842. Till 1865 all medicines had to be provided by the officer out of his salary, but after that year certain medicines such as cod-liver oil and quinine could be provided by the Guardians. It may be generally said that medicines are still supplied by medical officers out of their salaries, except in Unions which have established dispensaries.

The Metropolitan Poor Act, 1867, provided for the establishment of dispensaries throughout the Metropolis. Medicines were provided by the Guardians, a dispenser giving his whole time to the work, and the medical officer was seen there at a fixed hour every day. This system raised the standard and increased the efficiency of medical outrelief, while the great majority of serious cases were promptly despatched to the Poor-law infirmaries. Acting presumably under their general powers, the Central Authority authorised the establishment of dispensaries in a number of the large urban centres.

### Nourishment.

The medical officer has power to recommend extra nourishment being supplied to a sick person under his charge, and as a rule the Guardians give effect to such recommendations. Medical treatment consists quite as much in the administration of a sufficient and appropriate diet as in the exhibition of drugs, and treatment at home is difficult without the additional requisites and nourishment. There is a continual struggle on the part of medical officers and others to give medical extras as a part of relief, which is sometimes sought for the sake of such extras. The Central Authority consider it a description of relief which, as it has a constant tendency to increase, requires to be most carefully watched. In some cases there is an undesirable delay in supplying such extra nourishment.

### Nursing of Outdoor Sick Poor.

In 1892 the Central Authority issued an order empowering the Guardians to appoint district nurses. Only a few Unions have done so, but the order has had the indirect effect of stimulating subscriptions by the Guardians to Nursing Associations. There has been in recent years a remarkable expansion of voluntary effort for the provision of district nurses. The nurses in England and Wales on August 1, 1907, in connection with the Queen Victoria's Jubilee Institute for Nurses was 1,803, and of the nursing associations affiliated to the Institute 242 received annual grants from Boards of Guardians amounting to £3,632 15s.; associations affiliated to County Associations (employing about 493 village nurses) received grants amounting to £386 11s. At Chichester and Merthyr Tydfil the nursing staff of the workhouse has successfully been made available for district nursing.

### Medical Relief on Loan.

The Poor-law Commissioners recommended medical relief being given by way of loan. But owing to the difficulty of apportioning the charges for salary on each case, and local disinclination to restrict medical relief, the suggestion was not widely adopted, or was limited to classes of persons having over a certain amount per week. Often no attempt is made to recover the cost. In London the amount recovered goes to the Metropolitan Common Poor Fund, and there is not much inducement to individual Boards to collect their "loan." In some Unions recovery is fairly carried out, successfully so where the relieving officer collects the money in small instalments. It is said that granting it on loan has a tendency to prevent a second application, thereby deterring the honest who would wish to pay. If a small rate were fixed it would be apt to create an erroneous impression that the entire cost is being repaid. The objection to free medical relief is that it will interfere with provident societies and efforts to induce the poor to provide for themselves.

### Deterrence of Outdoor Medical Relief.

Various deterrents, such as appearance before the Guardians and limitation of the duration of the order, have been adopted. But in the opinion of some the greatest deterrent is in its association with the Poor Law, the decent poor waiting to the very last rather than apply. On the other hand there is a good deal of evidence that this association with the Poor Law has lost its terrors. The Medical Relief Disqualification Removel Act in 1885 was evidence of a change in public opinion, while the Old Age Pensions Act, 1908, by retaining poor relief as a disqualification and expressly excepting medical relief has further emphasised the distinction. The effect of the Act of 1885 on the number of Dispensary Orders in London was an immediate and steady rise to 1895, but a decline then set in, and the figures have since reached a point below the previous low-water mark.

### Agencies for Supplying Poor Persons with Medical Assistance Outside the Poor Law.

(1) *Voluntary Hospitals.*—In the ante-1867 period the paupers were sent to voluntary hospitals for operations and special treatment, as is the case to-day, especially where no separate infirmary has been provided. Voluntary hospitals have been greatly extended, but have been outstripped by Poor-law infirmaries. Taking nine large towns there were, in 1906, 15,365 beds in voluntary hospitals and 26,900 in Poor-law infirmaries, exclusive of over 10,000 beds in Metropolitan Asylums Board hospitals. The absence of or deficiency in voluntary hospital accommodation is being gradually but surely supplied by the infirmaries.

Voluntary hospitals in England and Wales in 1906 dealt with 245,513 in-patients and 1,492,423 out-patients. In London about 40 per cent. of the patients are of a similar class as applicants for Poor-law medical relief. But voluntary hospitals are largely taken advantage of by the well-to-do. At the Westminster Hospital in 1903-4-5 it was found that an average of 15 per cent. of the out-patients could have arranged their own treatment through a provident dispensary or Friendly Society.

(2) *Provident Dispensaries and Medical Clubs.*—Provident dispensaries existed before 1834, and the Commissioners did all in their power to encourage the formation of medical clubs. It is clear from the evidence that at the present time large numbers of the population provide for medical attendance in one form or other. As regards Friendly and other provident societies the direct provision of medical attendance forms only a subsidiary portion of their functions, the additional contributions for this benefit not usually being obligatory. Provident dispensaries sometimes cover as much as one in two of the population of towns. In rural districts the "medical club" is more prevalent. These are organised by several medical men or by one. In towns the medical club is very common; in certain districts "works clubs" cover almost the whole male working population. In seven towns—Coventry, Eastbourne, Hartlepool, Kidderminster, Norwich, Southampton, and Taunton, a "public medical service" has been organised among the doctors for treating the poorer working class on a provident basis. Sick benefit societies have frequently combined in one town, and appointed their own medical officer. By this means they secure the advantages of a provident dispensary, and the members' families are allowed to join. It is interesting to compare the membership of Friendly Societies with the number of paupers.

| | Friendly Societies. | Paupers. |
|---|---|---|
| 1803 ... | ... 704,350 ... | 1,039,716 |
| 1815 ... | ... 925,439 ... | 895,993 |
| 1904 ... | ... 5,700,000 ... | 932,000 |

Some Boards of Guardians have been very successful in starting and encouraging provident and medical clubs. Fees are from 1d. to 1½d. per week, and members of a provident dispensary as a rule have a choice of doctors, whose remuneration is either per visit or a fixed annual payment per member. The low rates of remuneration are in part due to competition among the doctors themselves, and, in towns, to the unfair competition of charitable agencies.

(3) *Free Dispensaries.*—In certain large centres free dispensaries exist. Some are old, endowed institutions; all are more or less dependent on voluntary subscriptions. The evidence clearly shows that they are abused by many who could well afford to pay ordinary medical fees, and by still more who are in a position to join a provident dispensary, thereby operating with hardship on the medical profession and having a distinctly pauperising tendency.

(4) *Sanitary Authorities.*—The Poor-law Commissioners soon realised that all infectious disease was accompanied by an immediate and ultimate charge on the poor rates, but it was not till the passing of the Sanitary Act in 1866 that the modern system of preventive medicine commenced. Sanitary authorities are already responsible for the treatment of a very high proportion of the total acute sickness among the poor. The line has never been strictly drawn between the prevention and treatment of disease, and under the Public Health Act, 1875, and the Public Health (London) Act, 1891, sanitary authorities may provide general hospitals for the sick inhabitants of their district. There is no limitation on the diseases to be treated or the class of persons to be benefited. Further, they may provide, subject to the sanction of the Local Government Board, a temporary supply of medicine and medical assistance for the poorer inhabitants. So far these powers have only been very slightly used.

The sanitary authority aims at prevention in the interest of the community; its work is almost invariably gratuitous. The Poor-law Medical Service aims at the cure of the individual who may be called upon for payment.

(5) *Education Authorities.*—Education authorities are required to send blind and deaf children to special institutions or provide such institutions, and are empowered to provide special classes or schools for physically or mentally defective children and epileptics. Under the Education (Administrative Provisions) Act, 1907, they have power to provide systematic medical inspection and supervision of school children and make such arrangements as may be sanctioned by the Board of Education for attending to the health and physical condition of the children. So far the Board has only called on the authorities to apply measures of amelioration to the ailments "erroneously regarded" as minor, but further advances are plainly indicated.

To sum up, the public provision of medical assistance, which in 1834 was entirely in the hands of the Poor-law authorities, is now shared between these authorities, the sanitary authorities and the education authorities.

### Review of the System of Medical Assistance to the Poor.

(a) *Inspection of Medical Relief by Central Authority.*

For the whole of England and Wales the Local Government Board have two Poor-law medical inspectors—a senior inspector, whose work is chiefly confined to London, and a junior inspector for the provinces. As there are some thousand Poor-law institutions in England and Wales it is obvious that a very large proportion of these only receive any medical inspection at long intervals. Medical officers, it is true, are called upon to report from time to time, but an officer can hardly be expected to invite criticism on an institution for which he is primarily responsible. Except in London the arrangements for outdoor medical relief have not been subjected to central inspection, either lay or medical. The primary responsibility of the Public Assistance Authorities for adequate inspection and supervision of the medical relief arrangements should be clearly recognised and carefully maintained. Meanwhile the Local Government Board's staff of medical inspectors should be increased.

(b) *Remuneration of Medical Officers—Indoor and Outdoor—and Supply of Medicines.*

There is a considerable body of evidence as to the want of uniformity in, and general inadequacy of, the salaries paid. The scale of fees allowed for operations was fixed so long ago as 1842, with considerable allowance for amputation and no provision for conservative surgery, and there is often difficulty in getting the Guardians to pay the fee for an assistant.

Where the salary paid is fairly adequate it is much diminished by the medical officer having to provide medicines for those under his charge. It is most objectionable that he should be placed in a position where his treatment of the case can be influenced by his ability or inability to supply certain necessary medicines. In future medicine should be directly provided by the Public Assistance Authorities, and in no case form a charge on the medical officer's remuneration. The actual amount and method of remuneration must be left to the proposed new authorities, but the salary should as far as possible be settled on a consideration of the Poor-law services concerned, and without taking into account any collateral appointment.

(c) *Institutional Medical Relief.*

1. *Accommodation for the Sick.*—Rural sick wards are frequently defective, but most of the objectionable conditions would yield to a comparatively small expenditure. The number of beds is generally equal to the demand at present. The structural arrangements of urban infirmaries—nearly all full to overflowing, are on the whole very good; but the Local Government Board should have more effective control over unnecessary expenditure. In a recent case the annual cost divided by the accommodation worked out at £85 per bed—*i.e.* 33s. per week.

2. *The Provision of Hospital Accommodation and Overlapping of Agencies.*—There is no system. Phthisical cases may in some areas be dealt with by the sanitary authority, and in others by the Poor-law authority; in others, again by both authorities. Sanitary authorities frequently provide isolation hospitals; in other places Poor-law institutions are the only ones available. Accident cases may be dealt with by the Poor-law, the sanitary, or the voluntary hospital authorities. Epileptic and other classes of children may be dealt with by the Poor-law, by the educational authorities, or by voluntary institutions. Transfers from voluntary hospitals to the infirmaries frequently take place. It has been suggested that co-operation should be on a medical basis—chronic cases to the infirmaries, acute and special cases to the hospitals—but no real interchange can take place so long as patients in Poor-law infirmaries, *ipso facto*, become paupers. Some more satisfactory organisation of medical charity is most desirable.

3. *Indoor Medical Attendance.*—As regards rural workhouses, this is, on the whole, adequately rendered. There is evidence in favour of the appointment of additional doctors for urban infirmaries, and strong representations have been received in favour of rendering the services of a staff of specialists and consultants available for infirmaries.

4. *Indoor Nursing.*—There is a difficulty of maintaining a sufficient staff in small rural workhouses, and some of these very small institutions are in constant danger of inefficiency, the only practical remedy for which is the formation of larger administrative areas. In urban infirmaries the nursing appears to be of a high standard, but perhaps hardly sufficient. The system of superannuation calls for revision, and the law as to the age limit (which is too high) should be altered.

5. *Classification of Sick Inmates.*—Separate institutions should be set apart for separate classes of the sick, more especially for the phthisical. Improvement might in the meanwhile be effected at moderate cost by making use of spare accommodation in rural workhouses suitably altered or arranged. But the only satisfactory method of securing efficient classification is to enlarge the administrative area and give the authority control over groups of institutions.

6. *Compulsory Detention of Sick Inmates.*—Persons suffering from infectious diseases which render them a danger to others can now be detained. Powers of detention require carefully safeguarding, and the knowledge that Poor-law authorities possessed such power would increase the objection to entering the infirmaries. The power of detention should be limited to a few well-defined classes of cases, and even then exercised as seldom as possible. The partially bed-ridden aged should only be detained if they are friendless or the responsible relatives acquiesce. With regard to phthisical cases, a beginning might be made with those most likely to communicate the disease or whose home conditions were such as to lead with practical certainty to infection. Venereal disease, with its sequelæ, is said to cause a large percentage of pauperism, and cases certified to be dangerous to others should be detained.

7. *Use of Poor-law Infirmaries for Purposes of Clinical Instruction.*—The main objection seems to be that patients resent being made the subject of a lecture. But the advantage to the medical profession and to the infirmaries far outweigh the possible objection which could easily be taken into consideration, and we recommend that infirmaries should be open for purposes of clinical instruction.

8. *Defective Records of Clinical Treatment.*—The medical officer should keep a "case paper," on which he should enter the medical details and other particulars in the manner usual in hospitals. Fuller records of treatment should be required also in the case of the outdoor sick. Such steps are indispensable to efficient control and supervision.

(d) *Outdoor Medical Relief.*

1. *Outdoor Medical Attendance.*—We are satisfied that the great majority of medical officers efficiently and punctually do their duties, and that many bestow on the poorer classes a considerable amount of attention for which they receive no recompense. But confidence in the doctor is an aid to recovery, and the poor, like the rest of us, have their own fancies. When the scheme, which will be detailed later, is in full working order, the member of a "provident dispensary" will be able to choose his own doctor from the list of those working with the dispensary. We recommend that certain cases in receipt of public assistance, such as the aged and widowed with young children, might be enrolled as members, and their fees paid by the Public Assistance Committee. To ensure prompt attendance in cases of sudden and urgent necessity, we think that every doctor who joins the dispensary should be required to give an undertaking to attend any such case for a suitable fee. We hope that, ultimately, it may be possible to dispense altogether with the service of the district medical officer, and that his duties will be shared among medical men practising in the district. Meanwhile arrangements might be made to retain the district medical officer on the staff of the provident dispensary.

2. *Outdoor Nursing.*—On the whole it may be said that adequate provision for the nursing of the outdoor sick poor has been made in only a small proportion of Unions. This is a serious defect, and has a prejudicial effect on the health of the paupers. The remedy must be sought in each locality, but existing and new associations ought to be encouraged, and the subscriptions paid by the authority should bear some relation to the monetary value of the services rendered. Indoor nurses in urban and rural districts might be utilised for outdoor cases; and failing these alternatives the authority should in populous districts maintain a staff of outdoor nurses. One of the first duties of the authority should be to organise a satisfactory and sufficient system of outdoor nursing, to be from time to time inspected by the Public Assistance Authority and the local board.

3. *Compulsory Removal of Sick Cases to Infirmary.*—We recommend that, subject to the same procedure as is adopted under the Health Acts, the relief authority should have power to remove compulsorily to an institution aged sick persons living alone and without relatives or friends to look to them, or persons suffering from a disease which cannot be properly treated at home, or which is a danger to the health of others living in the same house.

4. *Unconditional Outdoor Medical Relief.*—Outdoor medical relief, when granted, should be subject to conditions to be imposed by the authority, which conditions should include, *inter alia*, the maintenance of a healthy domicile and good habits.

(e) *Inaccessibility of Medical Relief and its Results.*

Medical relief is *normally* obtainable on an order from the relieving officer, instead of by direct access to a doctor. (It is not sufficiently known among the poor that overseers can grant emergency orders.) This leads to delay, and cannot be rigidly adhered to in country districts, while even in large centres of population the poor may not know where to apply. Doctors freely attend cases without an order, and many of them give their attendance and medicines, sometimes even extras or sustenance, and do not make a formal report to the relieving officer. It is estimated by Dr. McVail that 15 to 20 per cent. of the Poor-law cases receiving medical relief are not so recorded. We therefore recommend that medical assistance be more readily accessible to those who are in need of it.

(f) *Overlapping of Agencies Providing Non-institutional Medical Assistance.*

Except to a very limited extent there appears to be no real or effective co-operation between the Poor Law, the sanitary authorities, the education authorities, the out-patient departments of the voluntary hospitals, the free dispensaries, the voluntary dispensaries, the medical clubs and friendly societies. We recommend there should be co-operation, and shall submit proposals with that end in view.

As regards the out-patients' departments of voluntary hospitals, our special investigator found that, at three hospitals, 11 per cent. of the out-patients were actually in receipt of, or had within six months received, Poor-law relief in some form. It is for consideration whether the Public Assistance Committees should not relieve the hospitals of (*a*) those suffering from chronic ailments, and (*b*) those whose home conditions militate against the benefit of such treatment. Equally the out-patients' departments are being extended to the well-to-do and those sufficiently able to join a provident institution. We are convinced a strong effort should be made to circumscribe the work of out-patients' departments which should be used exclusively for casualties, consultations, and cases requiring expensive equipment, and recommend reorganisation on these lines.

Unless the work of education authorities in respect of the Act of 1907 is co-ordinated with that of the Public Assistance Authorities, the evils of overlapping will be accentuated. The Board of Education has recommended that the work be carried on in intimate conjunction with the public health authorities and under the direct supervision of the medical officer of health. The sanitary authorities would be responsible for the medical inspection of all children, and the Public Assistance Authority would provide the required treatment for all school children, so far as they required to be treated at the expense of the rates.

